# Etablierung und Optimierung einer routinemäßigen Resistenzbestimmung von *Helicobacter pylori* zur Förderung einer resistenzgerechten Eradikationstherapie

**DOI:** 10.1055/a-2645-6481

**Published:** 2025-09-11

**Authors:** Anke Hildebrandt, Reinhard Bornemann

**Affiliations:** 1 39549 Medizinische Klinik I, St. Vincenz-Krankenhaus, Datteln, Germany; 2 9185 Institut für Medizinische Mikrobiologie, Universität Münster, Münster, Germany; 3 Innere Klinik, Universitätsklinikum OWL, Bielefeld, Germany; 4 AG2 Bevölkerungsmedizin und Versorgungsforschung, Fakultät für Gesundheitswissenschaften, Universität Bielefeld, Bielefeld, Germany

**Keywords:** *Helicobacter pylori*, diagnostische Möglichkeiten, Eradikationstherapie, resistenzgerechte Therapie, Antibiotic stewardship, *Helicobacter pylori*, diagnostic options, eradication therapy, resistance-guided therapy, antimicrobial stewardship

## Abstract

In der Therapie einer *Helicobacter pylori* -Infektion lag der Schwerpunkt bislang auf empirischen Antibiotikaschemata. Angesichts zunehmender Resistenzdynamiken sowie regional unterschiedlicher Resistenzmuster erscheint jedoch eine Resistenzbestimmung bzw. resistenzgeleitete Therapie zielführender. Dies gilt nicht nur zur Steigerung der Effektivität einer individuellen Therapie, sondern auch im Sinne von Antibiotic stewardship. Insbesondere im Rahmen der überwiegend gastroskopischen Diagnostik stehen verschiedene Optionen der Resistenztestung mittels Kultur und PCR zur Verfügung. Daraus lassen sich individualisiert die jeweils optimalen resistenzgerechten Therapieschemata ableiten.

## Abstract (en)

The treatment of *Helicobacter pylori* infection, so far, focussed on empirical antibiotic regimens. Facing increasing resistance dynamics and regionally different resistance patterns, however, resistance testing respectively resistance-guided therapy appears to be more appropriate. This helds not only in order to increase the effectiveness of individual therapy, but also in the sense of antibiotic stewardship. In particular, in the context of predominantly gastroscopic diagnostics, various options for resistance testing using culture and PCR are available. Thus, the optimal resistance-oriented therapy regimens can be derived on an individual basis.

## Abkürzungen

AB: Antibiotika
ABS: Antibiotic stewardship
HP: *Helicobacter pylori*
HUT: *Helicobacter* -Urease-Test
PE: Probeexzision, Biopsie

## Hintergrund

Eine Infektion im Magen mit dem Bakterium *Helicobacter pylori* (HP) ist häufig Ursache von gastrointestinalen Beschwerden bzw. Erkrankungen: B-Gastritis, Ulcus ventriculi bzw. duodeni, atrophische Gastritis, intestinale Metaplasie, MALT-Lymphom und Magenkarzinom. Eine HP-Infektion erhöht das Magenkarzinom-Risiko um das 4–6 fache 1 , ca. 80% der Magenkarzinome weltweit sind HP-assoziiert 2 . In Deutschland liegt die HP-Prävalenz bei 35,3% (95 %, CI 31,2–39,4) 3 . Derzeit läuft eine große multizentrische Studie in Deutschland, u.a. zur HP-Prävalenz, deren erste Ergebnisse eine serologische HP-Prävalenz von 19,3% zeigen (wobei die konfirmierte Prävalenz bei positiver Serologie bei ca. 50% der Proben liegt) 4 . Mehrere Faktoren sind mit höherer Prävalenz bzw. Wahrscheinlichkeit einer HP-Infektion assoziiert: höheres Lebensalter, Migrationshintergrund und HP-Infektion bzw. -assoziierte Erkrankungen (v.a. Magenkarzinom) bei Familienangehörigen 3 . Das individuelle Risiko ist darüber hinaus u.a. abhängig von geografischer und ethnischer Zugehörigkeit, sozioökonomischen Bedingungen und dem Hygienestandard 3 .

Der Nachweis einer HP-Infektion bedingt im Regelfall die Einleitung einer sog. Eradikationstherapie mittels Antibiotika (AB). Die Indikationen hierzu ergeben sich prinzipiell aus der aktuellen Leitlinie der Dt. Ges. für Verdauungs- und Stoffwechselkrankheiten (DGVS) 3 . Dafür stehen mehrere Schemata mit jeweils unterschiedlichen AB-Wirkstoffkombinationen zur Verfügung, unter Einbezug der AB Amoxicillin (AMX), Clarithromycin (CLR), Levofloxacin (LVX) 1 , Metronidazol (MTZ), Rifampicin/Rifabutin (RIF) 2 und Tetrazyklin (TET).

Wie bei anderen Infektionen mit vermuteter bakterieller Genese kann entweder eine empirische Therapie erfolgen, unter Berücksichtigung des wahrscheinlichsten Spektrums von Resistenzen, oder es erfolgt vorab eine Resistenzbestimmung, um dann resistenzgerecht antibiotisch therapieren zu können. Ob primär eine Resistenzbestimmung erfolgt, hängt u.a. von der Schwere des Krankheitsbildes ab, bzw. von den Rahmenbedingungen etwa in der ambulanten Medizin. Bei HP ist eine Resistenztestung auf die diversen infrage kommenden AB-Wirkstoffe möglich. Die WHO führte Clarithromycin-resistente HP noch bis 2024 als bakteruielles Pathogen mit hoher Priorität für die Entwicklung neuer AB 5 6 .

Die DGVS-Leitlinie betont, dass „die prätherapeutische Resistenzlage von HP von großer therapeutischer Relevanz“ sei, und daher die Auswahl eines Therapieschemas die Wahrscheinlichkeit einer möglichen AB-Resistenz berücksichtigen möge. Aus diesem Grund empfiehlt sie als Erstlinientherapie eine sog. Quadrupeltherapie mit MTZ und TET sowie mit Bismuth und Protonenpumpeninhibitor (PPI). Bis 2022 bzw. vor dem Erscheinen der Neuauflage der Leitlinie war die bevorzugte Erstlinientherapie eine sog. Tripeltherapie, meist mit AMX + CLR + PPI („französisch“). Diese Kombination war gemäß Leitlinie nicht mehr angezeigt, da epidemiologische Untersuchungen für CLR zunehmende Resistenzraten beschrieben. Als Quelle dient u.a. eine europaweite Studie 7 , die für Deutschland im Jahr 2018 eine CLR-Resistenzrate von 21,4% angibt – bei allerdings nur n=85 Probanden. Berücksichtigung fand wohl auch die aktuellste deutsche Studie 8 , die allerdings für 2018 – bei n=1171 – nur eine CLR-Resistenzrate von 14,5% ausweist. International besteht Konsens, dass eine primäre Resistenz gegen einzelne AB-Wirkstoffe bis zum – arbiträr festgelegten – Level von 15% zu tolerieren ist um ein darauf basierendes empirisches Schema gründen zu können 9 .

Allerdings bestanden gleichzeitig auch hohe Resistenzprofile für MTX mit etwa 38,9% (ohne separate Angabe für D, dabei 85/1211 Proben aus D) 7 . In der erwähnten Studie zu Deutschland insgesamt 8 sowie in einer weiteren Publikation aus derselben Arbeitsgruppe zu einer ostdeutschen Teilregion 10 war MTZ hingegen nicht erfasst. Entsprechend konstatiert die Leitlinie, dass ein „Mangel an dokumentierten regionalen Resistenzdaten für Deutschland“ bestünde (die letzte umfangreichere Übersicht hierzu stammt von 2014 11 ).

Die Leitlinienautoren erkannten diesen Einwand höherer Resistenzraten für MTZ vs. CLR trotz fehlender etablierter genotypischer Resistenztestung für MTX auch, verwiesen jedoch darauf, dass eine „in vitro“ nachgewiesene Resistenz nicht notwendigerweise auch eine Resistenz „in vivo“ bedeuten müsse. Sie bezogen sich dabei auf eine Studie, welche bei europäischen Patienten mit oder ohne vorheriger Eradikationstherapie mit ca. 90% Eradikationserfolg ein gutes Behandlungsergebnis nach einer empirischen Quadrupeltherapie zeigte 12 . In dieser Studie stammten allerdings von den insgesamt 2100 Patienten gerade einmal n=35 aus Deutschland, ohne nähere regionale Zuordnung. Zudem wies die Studie die Behandlungsergebnisse dieser deutschen Subpopulation in der Studie nicht gesondert aus. Auf dieser Basis erscheint die alleinige Fokussierung auf die Quadrupeltherapie im deutschen Kontext unter mikrobiologischen und ABS-Aspekten durchaus diskutabel (wobei im Umkehrschluss das Fehlen sowohl flächendeckender als auch lokaler Daten allein kein Argument für den Wechsel der empirischen Therapieempfehlung gem. Leitlinie wäre).

Eine weitere Studie mit n.b. therapienaiven Patienten 13 verglich Bismuth-Quadrupel- vs. „concomitant“-Therapie (AMX, CLR, MTZ und PPI) und fand bei ersterer 100% vs. letzterer 92,5% Eradikationserfolg, trotz 19% (n=18/96) MTZ-Resistenz in der Gesamtstichprobe – wobei bei allen diesen 18 Patienten eine erfolgreiche Eradikation möglich war – und schlussfolgerte daraus, dass eine nachgewiesene MTZ-Resistenz keinen Einfluss auf den Eradikationserfolg habe. Dies ist auch bereits aus einer anderen großen europäischen Studie bekannt 14 . Dabei bleibt aber außer Acht, dass beide verglichenen Therapieregime noch mindestens ein weiteres AB beinhalteten bzw. der Therapieerfolg ggf. allein auf dieses zurückzuführen sein könnte. In diesem Zusammenhang gibt es bereits Erfahrungen aus Asien mit einer Dreierkombination aus Bismuth, TET und PPI 15 sowie einer dualen Therapie mit Amoxicillin und dem Kalium-kompetitiven-Säurehemmer Vonoprazan 16 . In Deutschland steht allerdings Bismuth nur als feste Kombination zur Therapie einer HP-Infektion bzw. nicht als Einzelsubstanz zur ggf. Zusammenstellung individueller Therapieschemata zur Verfügung.

Unterstützend hierfür ist, dass HP-Resistenzen gegen AMX, RIF und TET weiterhin vernachlässigbar sind (<1%) – auch nach bereits gescheiterten Therapien (NRZ, pers. Kommunikation). Für Bielefeld und das umgebende Ostwestfalen-Lippe zeigen Erhebungen der beiden auf HP-Resistenzen testenden großen Labore für die Jahre 2019–2024 folgende Empfindlichkeiten: 99–100% für die Wirkstoffe AMX, RIF und TET, 81–90% für LVX, 48–68% für CLR und nur 39–45% für MTZ (Labore Diamedes und Krone, pers. Kommunikation).

An dieser Stelle ist allerdings einschränkend zu bedenken, dass aus den vorgenannten Resistenzdaten meist nicht hervorgeht, wie groß der jeweilige Anteil der therapienaiven vs. der schon vortherapierten Patienten war – letztere nach primärem Therapieversagen mit zu vermutenden höheren Resistenzraten.

Anhand des zuvor Gesagten wollten wir systematisch die Optionen eines differenzierten diagnostischen und therapeutischen Procederes bei HP überprüfen. Dies geschah auch vor dem Hintergrund der in Westfalen-Lippe in jüngerer Zeit intensivierten Bemühungen um ABS, etwa in Form des ABS-Netzwerks Westfalen-Lippe 17 . Im Rahmen dieses Netzwerkes hatte sich 2022 eine Arbeitsgruppe speziell zum Thema HP gebildet, bestehend aus Ärztinnen und Ärzten aus dem Klinikum Bielefeld und dem St. Vincenz-Krankenhaus Datteln.

Zielstellung dieser Arbeit war es, zum einen die labortechnischen Möglichkeiten der Resistenztestung von HP nebeneinanderzustellen und zum anderen, bzw. darauf aufbauend, praxistaugliche Verfahrensweisen in Richtung einer resistenzgerechten HP-Eradikationstherapie zu entwickeln.

## Erreger- und Resistenztestung von HP

Zum Erregernachweis von HP per se stehen verschiedene Verfahren zur Verfügung 3 :

1. invasive Methoden (jeweils aus Magenbiopsien):
  - Histopathologie (als „Goldstandard“)
  - *Helicobacter* -Urease-Test (HUT)
  - Kultur
  - molekulargenetischer Nachweis (PCR)
2. nicht-invasive Methoden:
  - Stuhl-Antigen-Test (ELISA, Schnelltest)
  - ^13^ C-Harnstoff-Atemtest 3
  - IgG-AK-Nachweis im Serum (nicht zum Nachweis einer akuten Infektion bzw. nur für epidemiologische Studien geeignet)

Keine Testmethode ist für sich allein 100% spezifisch. Bei bereits niedrigen, und weiter sinkenden, Prävalenzen der HP-Infektion in den industrialisierten Ländern steigt das Risiko falsch-positiver Befunde. Dazu schreibt die DGVS-Leitlinie 3 : „ *Für eine zuverlässige H. pylori-Diagnostik sollten eigentlich zwei positive Testergebnisse mit unterschiedlichen Verfahren vorliegen. Im praktischen Alltag ist dies allerdings kaum zu vermitteln, auch ist es bei bestimmten Konstellationen nicht notwendig. Im Falle eines endoskopisch nachgewiesenen Ulkus duodeni genügt ein positiver H. pylori-Test für die Einleitung einer Eradikationstherapie. Auch der histologische Nachweis von H. pylori in Kombination mit einer chronisch aktiven Gastritis ist ausreichend.“*

Resistenzbestimmungen von HP sind mittels zwei verschiedener Konzepte möglich: zum einen über die „phänotypische“ Empfindlichkeitstestung nach kultureller Anzucht in der Mikrobiologie und zum anderen über „genotypische“ Empfindlichkeitstestung mittels Polymerasekettenreaktion (PCR) 18 – meist nach Aufbereitung zur histopathologischen Begutachtung in der Pathologie.

Für die HP-Kultur in der Mikrobiologie ist das Verbringen der Magenbiopsien unmittelbar nach Entnahme in ein geeignetes Transport- und Anzuchtmedium erforderlich (z.B. Portagerm pylori/PortPyl, bioMérieux, Deutschland). Die Bebrütungs- bzw. Untersuchungszeit beträgt i.d.R. bis zu 14 Tagen. Standardmäßig erfolgt eine kulturelle Resistenztestung gegen sechs verschiedene AB – gegen die o.g. AMX, CLR, LVX, MTZ, RIF und TET – z.B. mittels Epsilometer-(E-)Teststreifen.

Eine PCR kann sich an die Histopathologie anschließen 4 . Zudem ist die PCR auch aus einer in die Mikrobiologie eingesandten Probe möglich. Im Regelfall resultiert zunächst die Aussage, dass HP in der Probe enthalten ist – womit formal die von der Leitlinie geforderte Bestätigung eines HP-Befalls durch eine zweite Testmethodik erfüllt ist. In weiteren PCR-Schritten sind nun auch bestimmte mit AB-Resistenzen assoziierte Genmutationen nachweisbar, und zwar üblicherweise für AMX (pbp1- und/oder pbp3-Gen), CLR (23S rRNA-Gen), Fluorchinolone (gyrA-Gen) sowie MTZ (insbes. rdxA- sowie frxA-Gen; 9 18 ). Im Versorgungsalltag in Deutschland erfolgt eine genotypische Resistenztestung gegen CLR und Fluorchinolone – nicht jedoch gegen MTZ, weil die Qualität einer genotypischen Resistenzbestimmung bei MTX nicht anerkannt bzw. nicht etabliert ist 19 20 und keine kommerzielle Resistenztestung in Deutschland verfügbar ist (NRZ, pers. Kommunikation).

## Resistenzbestimmung im Kontext einer Gastroskopie

Die meisten HP-Nachweise bzw. daraus abgeleiteten Behandlungsindikationen dürften aus Gastroskopien bzw. aus Histologien hervorgehen. Bei der Histologie-Befundung ist allerdings die Gastroskopie schon beendet bzw. eine weitere Probeexzision, Biopsie (PE) zur ggf. mikrobiologischen Diagnostik nur durch eine erneute Gastroskopie möglich, was zu zusätzlichem Ressourcenverbrauch sowie zusätzlichen interventionsbedingten Risiken führen würde. Eine kulturelle Anzucht aus Histologiematerial ist nicht möglich, vor allem nach Einbringen der PE in Formalin bzw. aufgrund der unzureichenden Erregerbedingungen außerhalb seines natürlichen Habitats.

Zu überlegen wäre daher eine regelhafte PE-Gewinnung auch für die Mikrobiologie. Eine automatische mikrobiologische Versendung würde jedoch jeweils entsprechenden Aufwand und Kosten bedingen, wobei nur ein Teil der Patienten – je nach Kollektiv geschätzt etwa im Bereich von 20% – sich später in der Histologie als HP-positiv erweisen.

Alternativ könnte man eine zusätzliche PE für ggf. Mikrobiologie gewinnen, in ein geeignetes Medium einbringen (s.o.) und das Histologieergebnis abwarten. Nur falls dies positiv ausfällt, würde auch eine Mikrobiologie ausgelöst. Allerdings wäre dann eine zusätzliche Möglichkeit zur Aufbewahrung, ggf. Bebrütung und späteren Versende-Logistik erforderlich, die dann allerdings als Sammelversendung erfolgen könnte. Dieses Vorgehen erscheint jedoch in der Praxis aufgrund der logistischen Herausforderungen wenig praktikabel.

Möglich wäre auch eine Vorverlegung der „ob“-HP-Diagnose vor die Gastroskopie. Dies könnte nicht-invasiv in Form von Stuhl-Antigen-Tests oder ^13^ C-Harnstoff-Atemtests erfolgen. Jedoch erscheinen beide Optionen nicht praktikabel. Beim Stuhltest setzen die Umstände der Gewinnung einer Stuhlprobe und deren Abgabe in der Praxis und Weiterversendung ins Labor (evtl. auch direkt ins Labor), beim ^13^ C-Harnstoff-Atemtest eine nur an wenigen Orten vorhandene technische Logistik und einen extra Zeitaufwand des Patienten zum Aufsuchen der Teststelle voraus. Ferner hat man bei insbesondere stationären Gastroskopien oft keinen Vorlauf, da diese meist akut indiziert sind. Vorteil dieser Methode wäre jedoch die Möglichkeit einer bereits bei der Gastroskopie gewonnenen und danach eingesandten PE extra für die Mikrobiologie.

Eine weitere Option wäre die Anfertigung einer PCR nach stattgehabter Histologie, die jedoch das Risiko eines Sensitivitätsverlustes birgt. Hintergrund ist, dass in den Pathologien erschwerte genotypische Analysen durch formalinbedingte Kettenabbrüche generell auch für andere Infektionsnachweise (z.B. Tuberkulose) bereits etabliert sind. Das ist so nicht auf die mikrobiologischen Labore übertragbar, deren Anspruch in der Regel die kulturelle Anzucht der Erreger ist. Eine weitere ungeklärte Frage der Resistenztestung in der Histopathologie ist die daraus abzuleitende Therapieempfehlung. Pathologen interpretieren die genotypischen Analysen nicht und leiten daraus auch keine Empfehlungen ab. Zudem lässt eine PCR nur den Nachweis von Resistenzen auf CLR und Fluorchinolonen, nicht jedoch auf MTZ, zu. Aus diesem Dilemma heraus suchten wir nach praktikablen Alternativen.

Ausgangspunkt war die Vorgabe der aktuellen Leitlinie, dass eine ggf. HP-Infektion vorzugsweise mit zwei unterschiedlichen Verfahren zu belegen ist. In der Praxis erfolgt dies jedoch in der Regel mittels Histologie, bzw. ist der HUT nicht mehr regelhaft im Einsatz. An dieser Stelle entstand jedoch der Gedanke, aus einem zusätzlich durchgeführten HUT, falls dieser positiv ausfällt, möglicherweise eine Resistenztestung gewinnen zu können. Diesen Ansatz verfolgten wir in einer noch laufenden Machbarkeitsstudie, über die wir nach Abschluss berichten wollen.

**Abb. 1:**
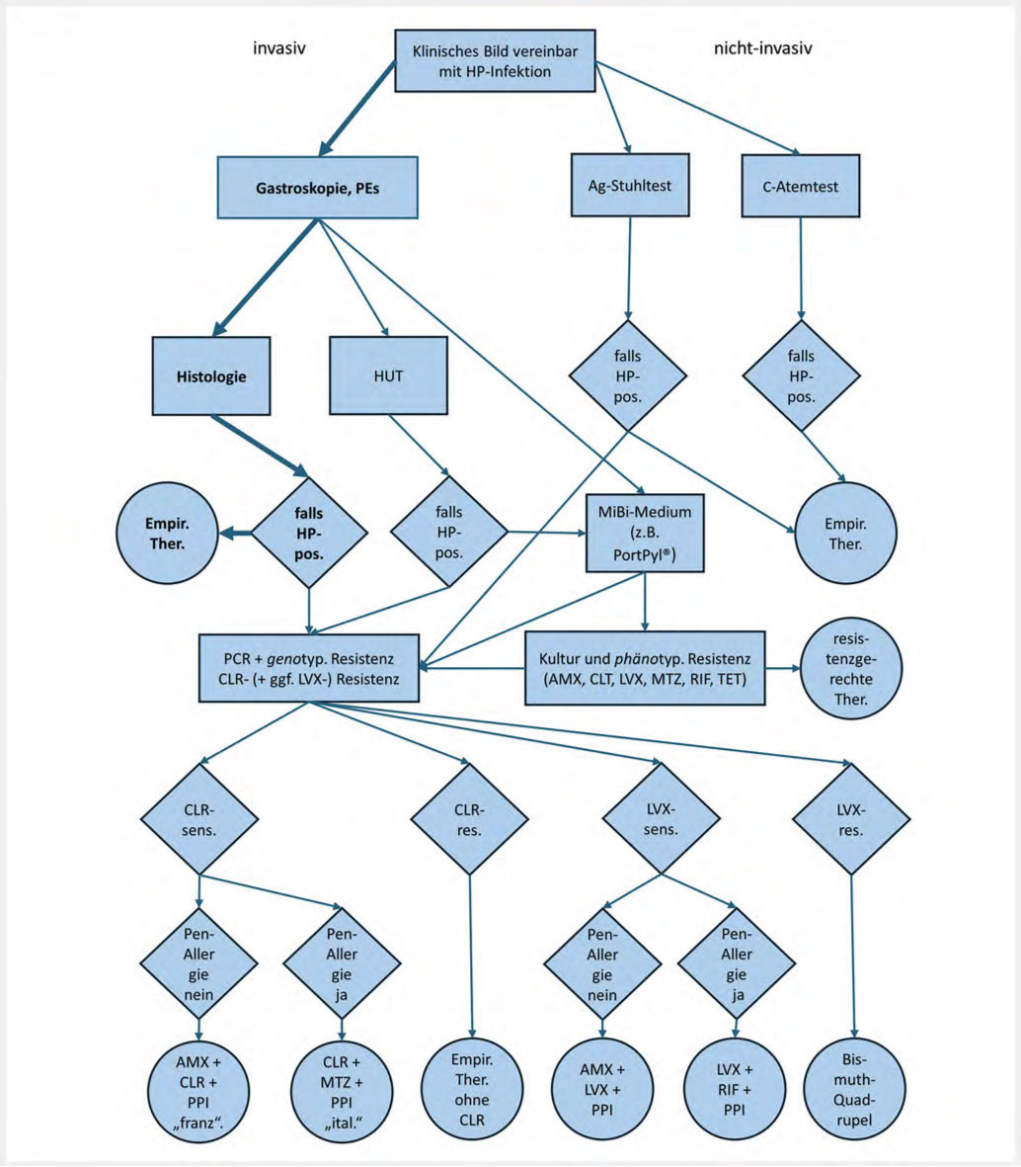
Ausgangspunkt ist ein Patientenkollektiv, bei dem eine HP-Testung prinzipiell infrage kommt. Je nach Setting erfolgt eine invasive oder nicht-invasive Diagnostik. Invasiver Diagnostikzweig: Der Standardfall ist hervorgehoben: Gastroskopie mit PEs, Histologie, und falls HP-pos. empirische (Bismuth-basierte Quadrupel-)Therapie (an dieser Stelle alternativ perspektivisch auch ein empirisches Schema gem. der lokalen Resistenzlage). Bei der Gastroskopie ist auch eine Entnahme von PEs optional für HUT und/oder Mikrobiologie via MiBi-Medium möglich. Falls der HUT HP-pos. ausfällt, kann sich entweder eine kulturelle Anzucht mit phänotypischer Resistenz und/oder eine PCR mit genotypischer Resistenz anschließen (wozu die Proben aus dem HUT vorher jeweils in ein geeignetes Medium zu überführen sind). Aus der kulturellen Anzucht ist (neben dem Erregernachweis per se) ein umfassendes HP-Resistogramm möglich, woraus eine komplett resistenzgerechte Therapie ableitbar ist. Via PCR erfolgt (neben dem Erregernachweis per se) zunächst eine Testung auf CLR-Resistenz: a) Falls CLR-sensibel, kann eine CLR-basierte Tripeltherapie erfolgen. Die Auswahl der CLR-basierten Tripeltherapie richtet sich sodann zunächst nach einem ggf. Vorhandensein einer anamnestischen Penicillin-Allergie: bei deren Nichtvorliegen > Schema mit CLR, AMX und PPI („französisch“), ansonsten CLR, MTZ und PPI („italienisch“). b) Falls CLR-resistent, kann wiederum gem. Leitlinie eine Bismuth-basierte Quadrupeltherapie erfolgen, bzw. alternativ ein Schema gem. der lokalen Resistenzlage (ohne CLR). Falls zusätzlich zur CLR- auch eine Fluorchinolon-PCR möglich ist: Falls Fluorchinolon sensibel ist, und keine anamnestische Penicillin-Allergie vorliegt, ist ein Schema mit (z.B.) LVX bzw. Moxifloxacin, AMX und PPI möglich; bei hingegen Penicillin-Allergie wäre eine Tripeltherapie mit LVX oder Moxifloxacin, Rifabutin und PPI möglich. Falls CLR- und Fluorchinolon-resistent, resultiert erneut eine Bismuth-basierte Quadrupeltherapie. Nicht-invasiver Diagnostikzweig: Falls ein HP-Nachweis mittels Stuhl-Ag-Test oder Atemtest erfolgt ist, resultiert entweder empirisch nach Leitlinie eine Bismuth-basierte Quadrupeltherapie, oder alternativ ein Schema entspr. der lokalen Resistenzlage; ggf. ist aus Stuhl-Ag auch eine PCR auf CLR-Resistenz möglich mit einer CLR-Resistenz (dann s.o.).

Unter Einbeziehung der verschiedenen in der Praxis eingesetzten diagnostischen Verfahren entwickelten wir schließlich einen nota bene explorativen Algorithmus, der die unterschiedlichen Optionen zur möglichst resistenzorientierten Diagnostik und Therapie enthält ( Abb. 1 ).

Die jeweiligen Vor- und Nachteile der empirischen vs. resistenzgeleiteten Eradikationstherapie anhand verschiedener Aspekte ergeben sich aus **Supplementtabelle S1** (im Online-Material).

## Diskussion

Die DGVS-Leitlinie von 2022 schlägt zur HP-Eradikation generell eine Bismuth-basierte Quadrupeltherapie vor. Diese erscheint aus mehreren Gründen nicht mehr angemessen. Im Zeitalter von ABS bzw. Diagnostic Stewardship (DGS) sollte eine AB-Therapie möglichst resistenzgerecht sein, um beim individuellen Patienten das bestmögliche Ergebnis zu erzielen bzw. therapieassoziierte Nebenwirkungen nur bei möglichst resistenzgerechter Therapie zu akzeptieren. Darüber hinaus verstärkt der Einsatz unwirksamer AB zusätzlich die Resistenzdynamik der Population. Vielmehr ist die Einheit von „Antibiotika-Resistenztestung, Resistenzsurveillance und ABS“ zu fordern. Es scheint in den aktuellen Empfehlungen einen Trend hin zur generellen Resistenztestung selbst vor einer Erstlinientherapie zu geben. Dabei weisen Malfertheiner et al. auch auf das damit verbundene Dilemma von ABS insbes. im ambulanten Bereich hin, dass nämlich solche Resistenztestungen immer mit zusätzlichem Aufwand und Kosten sowie nicht immer einfacher Verfügbarkeit bzw. Logistik verknüpft sind 9 .

Eine HP-Eradikation ist i.d.R. nicht eilbedürftig. Daher erscheint das Warten auf eine Resistenztestung (Kultur bis zu 14 Tage, PCR bis zu 7 Tage – je nach Logistik) bis zum Therapiebeginn durchaus akzeptabel.

Wir haben ein differenziertes Panel an potenziellen Alternativen zur HP-Resistenzbestimmung vorgelegt. Aus unserer Sicht ist eine Prüfung dieser Alternativen in breitem Umfang auf ihre jeweilige Umsetzbarkeit und Praktikabilität deutschlandweit in verschiedenen Versorgungskontexten, stationär und ambulant, erforderlich. Diese Alternativen beruhen einerseits auf einer phänotypischen Resistenztestung via Kultur und andererseits auf einer genotypischen Testung via PCR. Beide setzen eine invasive Diagnostik, bzw. PE-Gewinnung mittels Gastroskopie voraus. Während die Kultur Resistenzmuster für alle sechs in Frage kommenden AB liefert (AMX, CLR, LVX, MTZ, RIF und TET), liefert die PCR mit in Deutschland erhältlichen kommerziellen Systemen dies nur für CLR und Fluorchinolone. Damit wäre aus therapeutischer bzw. ABS-Sicht die Kultur erste Präferenz.

Allerdings setzt die Kultur eine zusätzliche Logistik voraus: separate PE-Gewinnung neben den obligaten PEs für die Histologie, gesondertes HP-Transportmedium, ggf. Versendung an ein Speziallabor, verbunden mit Extrawegen für Diagnostik-Anforderung und Befundrücklauf. Auch bedenkenswert ist der erhöhte Aufwand in der Endoskopie (Zeit, Materialverbrauch) und ein wenngleich geringes erhöhtes Blutungsrisiko durch zusätzliche PEs – in ex-ante-Unkenntnis, ob überhaupt eine HP-Infektion vorliegt.

Demgegenüber kann die PCR im Nachgang nach der Histologie erfolgen, aus bereits vorhandenem bzw. aufbereitetem PE-Material, mit allerdings ebenfalls etwaiger Versandlogistik an ein Speziallabor, sowie entspr. Kommunikation.

Bislang unzureichend berücksichtigt erscheint die Frage, ob HP-Eradikationsschemata, wie bisher, überhaupt zwei unterschiedliche AB benötigen, oder ob nicht ein einzelner AB-Wirkstoff ausreichend sein könnte – für den nota bene eine Sensibilität besteht. Das Konzept mit i.d.R. zwei AB-Wirkstoffen stammt aus der Zeit, als Eradikationstherapien regelhaft empirisch stattfanden. Hierzu wäre weitere Forschung nötig, mit dann allerdings auch zusätzlichem großem Potential für ABS, bei evtl. möglichem Verzicht auf ein AB in einer großen Zahl von Therapien deutschlandweit und darüber hinaus (ein Ansatz, den bereits einige Studien, in Kombination mit einer stärkeren Säurehemmung, verfolgen, vgl. z.B. 21 22 23 ).

Gemäß DGVS-Leitlinie „sollten eigentlich zwei positive Testergebnisse mit unterschiedlichen Verfahren vorliegen“, was zusätzlich für ein Testkonzept mit einer Ergänzung zur routinemäßigen Histologietestung spräche, entweder per HUT, Mikrobiologie oder PCR (ggf. auch per Ag-Stuhltest oder ^13^ C-Atemtest). Dies ist allerdings in der Praxis nicht regelhaft umsetzbar, bzw. ist bei der chronisch-aktiven Gastritis der alleinige histologische HP-Nachweis ausreichend.

Bezüglich der Optionen zur Resistenztestung spielt das Alter der Patienten eine Rolle: gem. DGVS-Leitlinie bzw. Maastricht-VI wird bei Patienten <50 Jahren ohne spezifisches Risiko bzw. ohne „Alarmsymptome“ eine nicht-invasive Diagnostik empfohlen. Da die Leitlinie zwei Tests empfiehlt, bedingt das die Kombination aus der ^13^ C-Atemdiagnostik und der Ag-Stuhldiagnostik. Nur bei der Ag-Stuhldiagnostik ist ggf. eine PCR-basierte Resistenztestung möglich, was folglich die Sensibilitätstestung für CLR bei dieser Altersgruppe und damit die Möglichkeiten einer resistenzgesteuerten Therapie einschränkt.

In der Praxis besteht das Problem, dass nur wenige Arztpraxen einen der beiden nicht-invasiven Tests anbieten können und dieser dann das Laborbudget deutlich belastet (pers. Kommunikation mit Kolleg:innen des Qualitätszirkels Vestnet e.V.). Eine häufig zum Einsatz kommende Alternative ist daher auch bei Patienten <50 Jahren mit dyspeptischen Beschwerden entgegen der Leitlinie eine Überweisung zur Gastroskopie und damit Durchführung einer invasiven Diagnostik. Es zeigt, dass die Umsetzung der Empfehlungen der Leitlinie in der Praxis mitunter auf Schwierigkeiten stößt.

Ein wichtiger Aspekt ist die Finanzierung einer routinemäßigen HP-Resistenztestung. Aus Sicht der mikrobiologischen Labore ist die HP-Diagnostik aktuell finanziell nicht attraktiv, was eine Ursache dafür ist, dass nur wenige Labore in Deutschland dies anbieten. Die HP-Anzucht ist aufgrund der besonderen Kultivierungsbedingungen des Bakteriums (Verwendung spezieller Nährmedien, langsames Wachstum, keine Verfügbarkeit einer Disk-Diffusionstestung aufgrund fehlender Breakpoints bzw. Fehlen einer automatisierten MHK-Bestimmung) eine kosten- und personalintensive Untersuchung. Die Spezialmedien erwerben die Labore in der Regel kommerziell. Gleiches gilt für die bei der kulturellen Resistenztestung zum Einsatz kommenden E-Test-Streifen. Die Kosten für eine PCR und genotypische Resistenztestung sind je nach Verfahren, Gensonde und der Anzahl der untersuchten Proben von 100–200 € möglich. Kommerziell verfügbare PCR-Tests haben die Entwicklung von kostengünstigeren in-house-Verfahren – nicht nur bei der HP-Diagnostik – aufgrund der Qualitätsstandards in den Hintergrund gedrängt. Dadurch haben Labore wenig Spielraum, die Kosten zu beeinflussen. Am Ende können die kulturelle und genotypische Resistenztestung zusammen einen Betrag von 200–300 € erreichen, den die Labore oft nicht kostendeckend abrechnen und diesen Verlust z.T. mit anderen Untersuchungen kompensieren.

Aufgrund der Unterfinanzierung der Resistenztestung besteht die Gefahr, dass die noch wenigen durchführenden Labore in Deutschland ihre HP-Diagnostik langfristig aufgeben. Das Referenzzentrum allein kann unsere Vision einer routinemäßigen HP-Resistenztestung vermutlich nicht bewältigen.

Aus Sicht der Untersucher, die ambulant und stationär Gastroskopien durchführen, erfordert eine HP-Resistenzdiagnostik zusätzliche Ressourcen verschiedener Art. Die Kosten der HP-Resistenztestung sind aktuell – ohne vorheriges Versagen einer empirischen Therapie – eine Eigenleistung der Einsender. Im stationären Bereich lohnt sich eine solche zusätzliche Diagnostik finanziell nicht, da deren Kosten nicht separat im DRG-System abbildbar sind. Hinzu kommt, dass die Patienten bis zum Ergebnis der Resistenztestung meist bereits in den ambulanten Bereich entlassen sind und die Therapie dann dort stattfindet. Krankenhäuser würden somit die Diagnostik für den ambulanten Bereich finanzieren, was aufgrund des zunehmenden Kostendrucks der Häuser der Häuser problematisch erscheint erscheint.

Ambulante Untersucher sind häufig gut an ein pathologisches Institut angebunden, mitunter weniger gut an ein mikrobiologisches Institut, sodass, neben den Kosten für die Diagnostik, die Logistik eine zusätzliche Hürde ist. Eine orientierende Umfrage in gastroenterologischen Bielefelder Praxen ergab, dass man zusätzlichen Resistenztestungen positiv gegenüberstünde, allerdings nur bei Vergütung der Zusatzkosten. Eine solche Erstattung ist gem. Rückfrage bei der Kassenärztlichen Vereinigung Westfalen-Lippe (KVWL) aktuell jedoch nicht vorgesehen. Hinzu kommt – wie oben bereits beschrieben – dass selbst die HP-Resistenzdiagnostik im laut Leitlinie berechtigten Fall (nach Therapieversagen) unterfinanziert ist. Es ist daher denkbar, dass in der klinischen Praxis beim Therapieversagen nach Quadrupeltherapie zunächst eine weitere empirische Therapie zum Einsatz kommt, obwohl die Leitlinie an dieser Stelle eine resistenzgerechte Therapie nahelegt.

Ein zusätzliches positives Resultat einer routinemäßigen HP-Resistenztest-Strategie wäre die Gewinnung von flächendeckenden HP-Resistenzmustern, die dann über die optimierte individuelle AB-Therapie hinaus in denjenigen Fällen dienen könnten, in denen die Durchführung einer Resistenztestung warum auch immer nicht möglich ist. Bislang erfolgt die Erhebung deutschlandweiter HP-Resistenzdaten nur im Rahmen der bereits erwähnten HelicoPTER-Studie 4 , die das NRZ koordiniert und die zum Ziel hat, im Laufe mehrerer Jahre 20000 Patienten in mehreren großen Studienorten einzuschließen. Dies ist ein guter Anfang, verfolgt jedoch nicht das langfristige Ziel einer dauerhaften Resistenzüberwachung auf nationaler und lokaler Ebene.

Ein möglicher Lösungsansatz wäre aus unserer Sicht, die Kosten der einzelnen Akteure im Gesundheitssektor nicht separat zu betrachten (Kosten der klinisch tätigen ambulanten und stationären Ärzte und Kliniken, Kosten der Labore). Vielmehr sollten die Kostenträger ein Interesse daran haben, eine routinemäßige HP-Resistenztestung unter gesamt-gesundheitsökonomischen Aspekten zu betrachten. So könnte eine resistenzgerechte Therapie gleich zu Beginn ein potenzielles Therapieversagen mit der Folge einer erneuten invasiven Diagnostik verhindern und damit Folgekosten sparen.

Abschließend ist noch darauf hinzuweisen, dass sich diese Bearbeitung auf eine erwachsene Patientenklientel bezieht, bzw. pädiatrische Klientele einer gesonderten Betrachtung bedürfen.

Eine orientierende Recherche im Januar 2025 in vergleichbaren Leitlinien bzw. Empfehlungen von ausgewählten europäischen Nachbarländern ergab:

- In Belgien liegt das primäre Augenmerk auf lokalen Resistenzdaten, zunächst für CLR. Die belgischen Empfehlungen favorisieren bei CLR-Resistenzen <15% eine CLR-basierte Therapie und bei CLR-Resistenzen >15% eine Empfindlichkeitstestung (anzumerken ist, dass auch in Belgien die MTZ-Resistenzen deutlich über den CLR-Resistenzen liegen) 24 .
- Eine dänische Arbeitsgruppe empfiehlt keine routinemäßige Empfindlichkeitstestung ( 25 ; sowie Bytzer, pers. Komm.).
- In den Niederlanden gibt es nach unserer Recherche keine nationale HP-Leitlinie; eine jüngere Arbeit fordert, dass die HP-Behandlung auf einem individuellen Resistenzprofil sowie in Übereinstimmung mit ABS-Prinzipien erfolgen solle 26 .
- Für Österreich fanden wir eine Empfehlung der Österreichischen Ges. für Gastroenterologie und Hepatologie, die als bevorzugte Therapie Bismuth-Quadrupel benennt, und dahinter eine Kombination aus AMX, CLR und MTZ sowie PPI („concomitant“) 27 .
- Eine Schweizer Empfehlung schließlich kritisiert generelle empirische Therapiestrategien und fordert stattdessen, dass „künftig auch die HP-Therapie gemäß den Prinzipien von ABS erfolgen soll bzw. AB nur noch resistenzgerecht zum Einsatz kommen sollen“ 28 .

## Schlussfolgerungen

Vor einer HP-Eradikationstherapie sollte eine routinemäßige HP-Resistenztestung erfolgen, um eine resistenzgerechte Therapie, im Sinne des individuellen Patienten wie auch von ABS, zu gewährleisten.

Zur HP-Resistenzbestimmung stehen unterschiedliche Verfahrensweisen zur Verfügung, aus denen sich die Untersucher im ambulanten bzw. stationären Setting je nach Verfügbarkeit und Praktikabilität die geeignetste wählen sollten.

Durch routinemäßige HP-Resistenztestung können in relevantem Umfang CLR-basierte Eradikationsschemata wieder zum Einsatz kommen, die zuvor aus epidemiologischen Überlegungen ausgeschlossen waren.

Eine Resistenztestung benötigt 1–2 Wochen zusätzlich; eine entspr. Therapieverzögerung erscheint jedoch klinisch ohne Weiteres vertretbar und trifft bei Behandlern wie bei Patienten meist auf Akzeptanz.

Ein kontinuierliches regionales Resistenzmonitoring im Rahmen einer regelhaft resistenzgeleiteten Therapie ist auch für weiterhin durchgeführte empirische Eradikationstherapien sehr nützlich.

### Ausblick

In Bielefeld und Datteln läuft seit 2023 eine multizentrische Studie zur Erprobung der aufgezeigten unterschiedlichen Varianten zur HP-Resistenztestung 29 . Eine Publikation erster Ergebnisse ist im Laufe des Jahres 2025 geplant.

## Footnotes

1 Levofloxacin (LVX) ist die in der Kultur getestete Leitsubstanz der Fluorchinolone; die PCR testet i.d.R. die generelle Fluorchinolon-Empfindlichkeit, diese umfasst dann u.a. LVX und Moxifloxacin.

2
Rifampicin ist die Testsubstanz im E-Test; Rifabutin ist Bestandteil einer Tripeltherapie mit Fluorchinolon + PPI zur Zweitlinientherapie, wenn eine anamnestische Penicillinallergie besteht (mit Vorbehalten, 3 ).

3
Die Tests ^13^ C/ ^14^ C sind technisch vergleichbar; da ^14^ C ein radioaktives Isotop ist, kommt in Deutschland nur noch ^13^ C zur Anwendung.

4 Ist die Histologie HP-positiv, erfolgt unter dem Mikroskop eine Markierung desjenigen Areals auf dem Objektträger mit der höchsten Bakteriendichte; nach Ablösen vom Objektträger erfolgt dann die PCR.

## Zusatzmaterial

Supplementary Material Zusatzmaterial
