# Supplementary material for: Establishment and optimization of a routinely resistance testing of Helicobacter pylori to encourage a resistance-guided therapy
Source: Z Gastroenterol. 2025 Sep 11;63(9):922–9. [Article in German] doi: 10.1055/a-2645-6481 (PMC12425596; doi:10.1055/a-2645-6481)
Supplement: Supplementary file 1 — Supplementary Material [file 10-1055-a-2645-6481_26545605.pdf]

Supplementtabelle S1: Vergleich der empirischen und resistenzgeleiteten Therapie von *H. pylori* anhand Diagnostik- und Therapie-relevanter Aspekte

**Relevanter Aspekt**	**Empirische Therapie***		**Resistenzgeleitete Therapie**	
	Pro	*Contra*	*Pro*	*Contra*
Praktikabilität	– kein Zusatzaufwand<br>– bei Patienten < 50 Jahre keine Endoskopie erforderlich	nur ein in D verfügbares Quadrupel-Kombinations-präparat<br>mit zeitweise<br>Lieferengpass		– i.d.R. Endoskopie erforderlich<br>– Zusatzaufwand durch Resistenzbestimmung<br><u>Kultur:</u><br>– nicht flächendeckend verfügbar, oft separate Logistik<br>– Transportmedien mit beschränkter Haltbarkeit, kühle Lagerung bis zur Verwendung erforderlich<br><u>PCR:</u><br>– nicht flächendeckend verfügbar (sowohl via Mikro-biologie als auch Pathologie)
Leitlinienadhärenz	Einheitlichkeit der Therapie durch standardisiertes Schema			Unsicherheiten durch Therapievarianz; Erfahrung und ggf. Beratung erforderlich
Personalisierte Therapie		nicht gegeben	gegeben	
Compliance		eingeschränkt bei 4 x 3 Tabl. pro Tag über 10 Tage (insges. 120 Tabl.)	Tripeltherapie: 2 x 2 Tabl. pro Tag über 14 Tage (insges. 56 Tabl.)	
Therapieeffizienz	prinzipiell niedriger vs. resistenzgeleiteter Therapie		– prinzipiell höher vs. empirischer Therapie	

			– Verringerung von Therapieversagen und damit erneuter invasiver Diagnostik	
Nebenwirkungen, Sicherheit		mehr NW durch Quadrupeltherapie		– erfordert invasive Diagnostik<br>– zusätzliche Biopsien mit ggf. erhöhtem Blutungsrisiko (Ausnahme: PCR durch Stuhl)
Mikrobiologische Aspekte	keine *in vitro*-Resistenzen gegen Bismuth bekannt	MTZ-Resistenzen in der Quadrupeltherapie ggf. ohne signif. Einfluss auf den Eradikationserfolg	– genotypische Resistenztestungen an (fixierten) Biopsien, HUT-Proben und Stuhl (nicht-invasiv) möglich	– genotypische R-Testung in D i.d.R. nur für CLR und Fluorchinolone möglich<br>– phänotypische Resistenztestung: spezielles Transportmedium und Spezialmedium erforderlich, nur R-Testung mit E-Teststreifen möglich, keine automatisierte R-Testung<br>– z.T. unklare Korrelation zwischen genotypischer und phänotypischer Resistenz<br>– nicht flächendeckend verfügbar<br>– Dauer bis zu 14 Tagen
Procedere bei Therapieversagen	nach der „Erstlinie“ schematische Alternativen für weitere Linien verfügbar	Nichterkennen von Mehrfachresistenzen, damit Risiko von mehrfachem Therapieversagen		prinzipiell erneute invasive Diagnostik erforderlich
Kosten der Diagnostik	keine zusätzlichen			– evtl. zusätzliche Kosten durch die Endoskopie (Patienten < 50 Jahre)

				– ggf. verlängerte Endoskopie-dauer durch zusätzliche PE für die Mikrobiologie<br>– zusätzlicher Zeitaufwand zur Gewinnung von PEs sowie für Versandlogistik<br>– Kosten für zusätzliche Medien und Versandmaterial<br>– Kosten der zusätzlichen Diagnostik mit Kultur bzw. PCR<br>– HP-Resistenzdiagnostik bislang unterfinanziert
Therapiekosten individuell		Quadrupel-Kombinations-präparat ca. 120 €	Tripeltherapie jeweils als Einzelpräparate ca. 20-40 €	Tripeltherapie-Kombinations-präparat dito ca. 120 €
Therapiekosten gesamt-gesundheits-ökonomisch	Kostenersparnis der Resistenztestung (aktuell unterfinanziert)	erhöhte Gesamttherapie-kosten im Fall eines Therapieversagens, was dann eine erneute Diagnostik und Therapie nach sich zieht	Kostenersparnis für das Gesundheitssystem:<br>– Tripeltherapie (wenn möglich) ist günstiger als Quadrupeltherapie<br>– Kostenersparnis durch weniger Therapieversagen und damit weniger Re-Gastroskopien<br>– Senkung des Resistenz-drucks	erhöhte Kosten in der Umsetzung der Resistenztestung, da nicht flächendeckend verfügbar und unterfinanziert
ABS-Tauglichkeit		– zunehmende AB-Resistenzen weltweit und in Deutschland<br>– auch durch Zunahme der Verordnung	– entspricht ABS (wo AB indiziert, vorzugsweise resistenzgerecht auswählen)	

		unwirksamer AB-Substanzen	– CLR-basierte Tripeltherapie (z.B. „französisch“) falls sensibel auch in Regionen mit sonst hohem CLR-Resistenzniveau	
Epidemiologische Aspekte		– liefert keine epidemiologischen Daten zur Resistenzsituation<br>– Resistenzraten gegen CLR, MTX und Fluorchinolone weltweit steigend<br>– empirische Therapie lässt ggf. Resistenzausbreitung unbemerkt voranschreiten<br>– erhöht ggf. Resistenzraten anderer bakterieller Erreger	– liefert epidemiologische Daten zur Resistenzsituation und darüber hinaus zur Surveillance eines sich ggf. verändernden Resistenzgeschehens<br>– wirkt der weltweiten Resistenzausbreitung entgegen	
Sonderfälle ohne invasive Diagnostik	– für dyspeptische Patienten < 50 Jahre nicht-invasive Diagnostik ausreichend<br>– im positiven Fall gem. Leitlinie empirische Therapie empfohlen			für dyspeptische Patienten < 50 Jahre mit nicht-invasiver Diagnostik Resistenztestung kaum möglich

* Soweit nicht anders erwähnt jeweils bezogen auf die in Deutschland empfohlene Bismuth-basierte Quadrupel-Erstlinien-Therapie
Abkürzungen: Tabl. = Tablette, PE = Probeexzision, Biopsie
